# Mechanism-based modeling of time-varying magnetic fields effects on cortical activity

**DOI:** 10.1186/1471-2202-15-S1-P116

**Published:** 2014-07-21

**Authors:** Julien Modolo, Alex W Thomas, Alexandre Legros

**Affiliations:** 1 Human Threshold Research Group, Lawson Health Research Institute, London, ON, N6A4V2, Canada; 2 Department of Medical Biophysics, Western University, London, ON, Canada; 3 Department of Medical Imaging, Western University, London, ON, Canada; 4 School of Kinesiology, Western University, London, ON, Canada

## Background

Understanding how extremely low-frequency (ELF, < 300 Hz) magnetic fields (MF) interact with human brain activity is an important question, especially regarding potential effects of power-lines MF (60 Hz in North America). Such knowledge is critical to 1) contribute to guidelines protecting public and workers from exposure to ELF MFs [1,2]; and 2) design novel non-invasive brain stimulation techniques using ELF MFs to interfere with pathological brain activity patterns.

## Methods

We used an extensively validated neural mass model [3] describing the main neuronal populations forming a cortical column, which we extended by including 1) a time-dependent membrane potential perturbation caused by the induced electric field; 2) a model linking post-synaptic calcium concentration and synaptic plasticity processes [4]. We used increasing levels of MF flux density at 60 Hz to identify the threshold for significant effects on simulated EEG alpha (8-12 Hz) power. A 4x3x2 ANOVA for repeated measured measures was conducted on EEG alpha power before/during/after exposure, with/without 60 Hz MF exposure, with/without synaptic plasticity.

## Results

Simulated EEG alpha power decreased with increased 60 Hz MF flux density (significant for 250<dV<500 μV when only pyramidal neurons were modulated), without significant effects from synaptic plasticity processes. If slow inhibitory interneurons [3] were also modulated, EEG alpha power decrease due to MF exposure was significantly diminished (see Figure).

**Figure 1:**
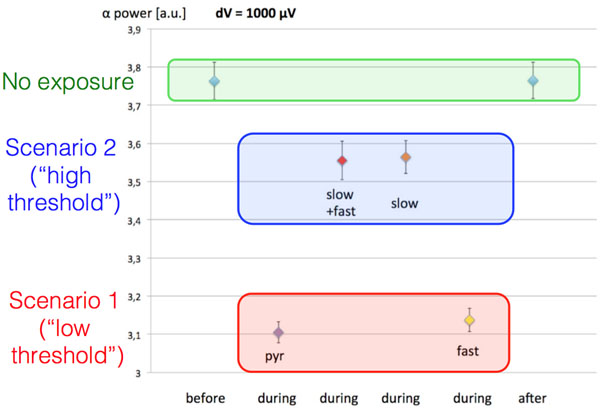


## Conclusions

The model will be used to 1) understand human data currently acquired in our group [5]; and 2) study *in silico* effects of transcranial alternating current stimulation and magnetic stimulation (tACS/TMS). Future work will include frequency-dependent effects from extracellular medium dielectric properties, and selective modulation of specific neuronal populations.
